# Discovery of Novel c-Met Inhibitors Bearing a 3-Carboxyl Piperidin-2-one Scaffold

**DOI:** 10.3390/molecules19022655

**Published:** 2014-02-24

**Authors:** Wei Zhang, Jing Ai, Dakuo Shi, Xia Peng, Yinchun Ji, Jian Liu, Meiyu Geng, Yingxia Li

**Affiliations:** 1 School of Pharmacy, Fudan University, Shanghai 201203, China; 2 Division of Antitumor Pharmacology, State Key Laboratory of Drug Research, Shanghai Institute of Materia Medica, Chinese Academy of Sciences, Shanghai 201203, China; 3 School of Pharmacy, Ocean University of China, Qingdao 266003, China

**Keywords:** c-Met, synthesis, kinase inhibitor, 3-carboxypiperidin-2-one

## Abstract

A series of compounds containing a novel 3-carboxypiperidin-2-one scaffold based on the lead structure BMS-777607 were designed, synthesized and evaluated for their c-Met kinase inhibition and cytotoxicity against MKN45 cancer cell lines. The results indicated that five compounds exhibited significant inhibitory effect on c-Met with IC_50_ values of 8.6−81 nM and four compounds showed potent inhibitory activity against MKN45 cell proliferation, with IC_50_s ranging from 0.57−16 μM.

## 1. Introduction

c-Met kinase is a transmembrane receptor tyrosine kinase (RTK). Upon binding of its endogenous ligand hepatocyte growth factor (HGF, also known as scatter factor, SF), c-Met receptor undergoes dimerization and in turn triggers signal transducers to mediate a variety of cellular responses such as cell growth, invasion, migration and survival [1,2]. The normal c-Met/HGF pathway plays an important role in embryogenesis and wound healing, but aberrant forms of this pathway (for example, as a result of overexpression of c-Met and HGF) have frequently been observed in a variety of human solid tumors and hematologic malignancies. Importantly, both increased levels of c-Met and HGF have been associated with poor clinical outcomes [3,4,5]. Therefore, c-Met has been pursued as an attractive anticancer drug target for the past two decades [6,7]. Several approaches to inhibition of the HGF/c-Met pathway in cancer cells have been reported, such as antagonistic ligands to c-Met, antibodies against HGF or c-Met, and small molecule c-Met inhibitors [8,9,10].

**Figure 1:**
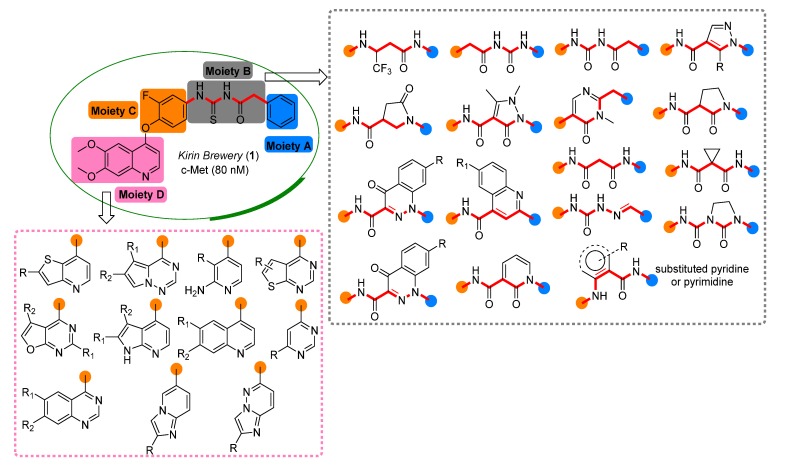
Representative scaffolds used for the structural optimization of c-Met inhibitors.

During the development of small molecular c-Met kinase inhibitors, a compound disclosed by Kirin Brewery Company in 2003 [11] could be regarded as a milestone (Figure 1). Structurally, this compound (**1**) is composed of four moieties: a phenyl group (moiety A), a bridge moiety B, an ortho-fluoro phenol and a 6,7-dimethoxyquinoline. Initiated by this discovery, numerous c-Met kinase inhibitors bearing diverse chemical scaffolds have been reported. Generally, structural optimization based on compound **1** mainly focused on moiety D and B. Replacement of the 6,7-dimethoxy- quinoline moiety by various *N*-containing heterocycles, such as substituted quinoline [12], thienopyridine [13,14,15], pyrrolopyridine [16], aminopyridine [17], thienopyrimidine [18], furopyrimidine [18], imidazopyridine [19] or imidazopyridazine [19], has been investigated. The bridge moiety B connecting moiety A and C was designed as linear [20,21,22] or cyclic [14,15,23,24,25,26], bearing at least one amide bond with 5-atoms in the main chain [22,24] (i.e., six chemical bonds distance between moiety A and C, Figure 1). However, there are little changes to moiety A and C, except for phenyl ring or substituted phenyl ring modifications to the former.

**Figure 2:**
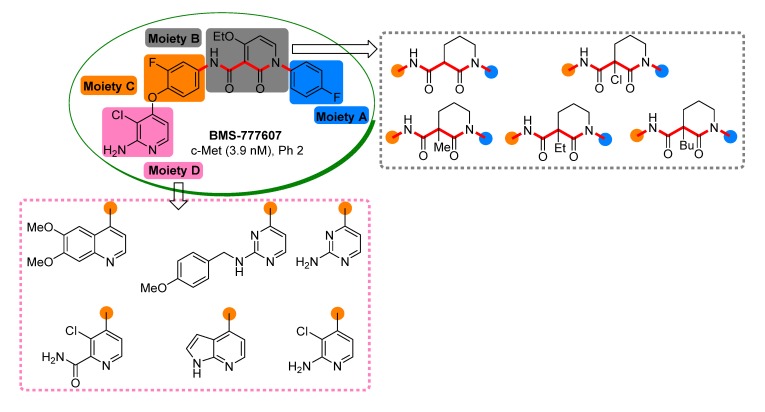
Scaffolds used for the structural optimization based on BMS-777607 in this paper.

A good example for these inhibitors is BMS-777067, which is now in phase 2 trial because of its excellent *in vivo* efficacy and favorable pharmacokinetic and preclinical safety profiles [17]. Taking BMS-777607 as leading compound, the design and synthesis of new derivatives with novel structures are under study in our laboratory. Preliminary investigation indicated that 3-carboxypiperidin-2-one is a promising scaffold for the design of new c-Met inhibitors. Herein we would like to report our efforts in this respect (Figure 2).

## 2. Results and Discussion

### 2.1. Chemistry

**Scheme 1:**
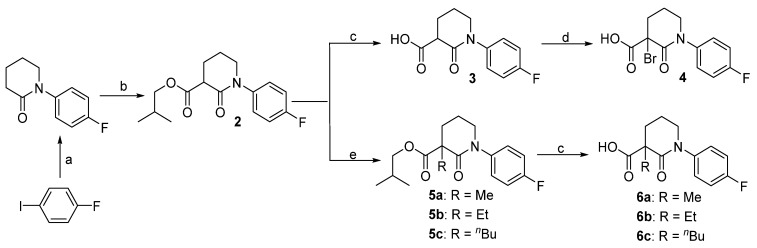
Synthesis of the piperidinone 3-carboxylic acids **3**, **4** and **6a**–**c**.

As shown in Scheme 1, saponification of isobutyl ester **2** with lithium hydroxide gave the piperidinone 3-carboxylic acid **3**, which could be further brominated giving compound **4** in 92% yield. On the other hand, deprotonation of compound **2** with sodium hydride, followed by treatment with an alkyl halide (MeI, EtBr, or *n*-BuBr) led to the corresponding α-substituted piperidinones. Saponification of these esters **5a**–**c** gave the corresponding carboxylic acids smoothly. In this way, we had five carboxylic acids (compounds **3**, **4**, **6a**–**c**) in hand, which would be used in next coupling step.

*Reagents and conditions*: (**a**) piperidin-2-one, CuI, K_3_PO_4_, DMF, 90%; (**b**) t-BuLi, isobutyl chloroformate, 82%; (**c**) LiOH, THF/MeOH/H_2_O, 87% for **3**, 88% for **6a**, 79% for **6b**, 84% for **6c**; (**d**) Br_2_/Et_2_O, 92%; (**e**) NaH/MeI, 87% for **5a**; NaH/EtBr, 76% for **5b**; NaH/*n*-BuBr, 83% for **5c**.

**Scheme 2:**
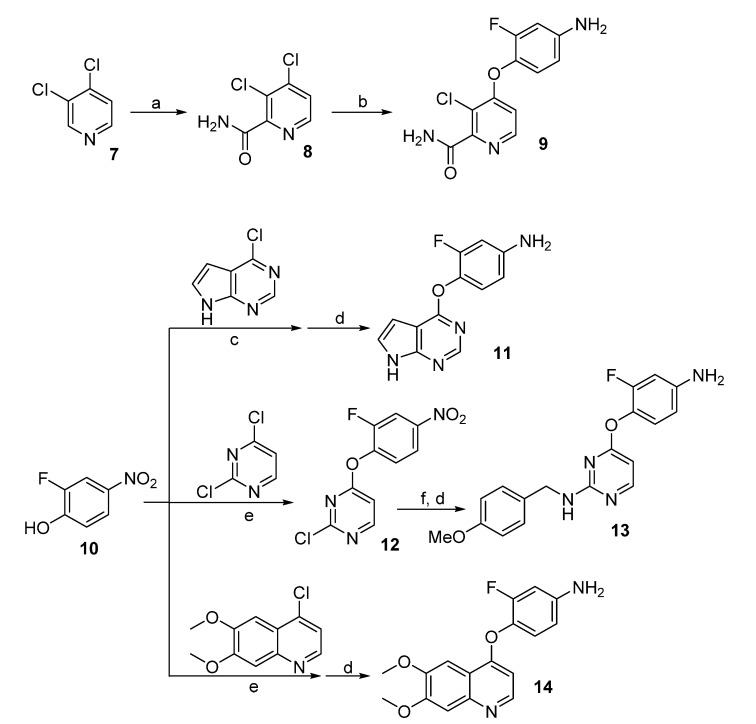
Synthesis of aromatic amines **9**, **11**, **13** and **14**.

Deprotonation of 3,4-dichloropyridine (**7**) with lithium 2,2,6,6-tetramethylpiperidide (TMPLi) followed by treated with trimethylsilyl isothiocyanate and acidic workup, gave 3,4-dichloropicolinamide (**8**) in 40% yield (Scheme 2). This pyridyl chloride was coupled with 4-amino-2-fluorophenol in the presence of potassium *tert*-butoxide to afford the aromatic amine **9** in 72% yield. Similarly, coupling of phenol (**10**) with 4-chloro-7*H*-pyrrolo[2,3-*d*]pyrimidine and 4-chloro-6,7-dimethoxyquinoline followed by conversion of the nitryl to an amino group gave amines **11** and **14**, respectively. For the preparation of substituted pyrimidine **13**, the amino group was introduced to the C-2 position before zinc-mediated reduction. Thus we had four aromatic amines (compounds **9**, **11**, **13** and **14**) in hand, which were subjected to the next step directly.

*Reagents and conditions*: (**a**) TMPLi, trimethylsilyl isothiocyanate, −78 °C, 40%; (**b**) *t*-BuOK, 4-amino-2-fluorophenol, 72%; (**c**) PhBr, 130 °C, 85%; (**d**) Zn, NH_4_Cl, 88% for **11**, 69% for **13**; 91% for **14**; (**e**) K_2_CO_3_, DMF; (**f**) 4-methoxybenzylamine, K_2_CO_3_, 68%.

**Scheme 3:**
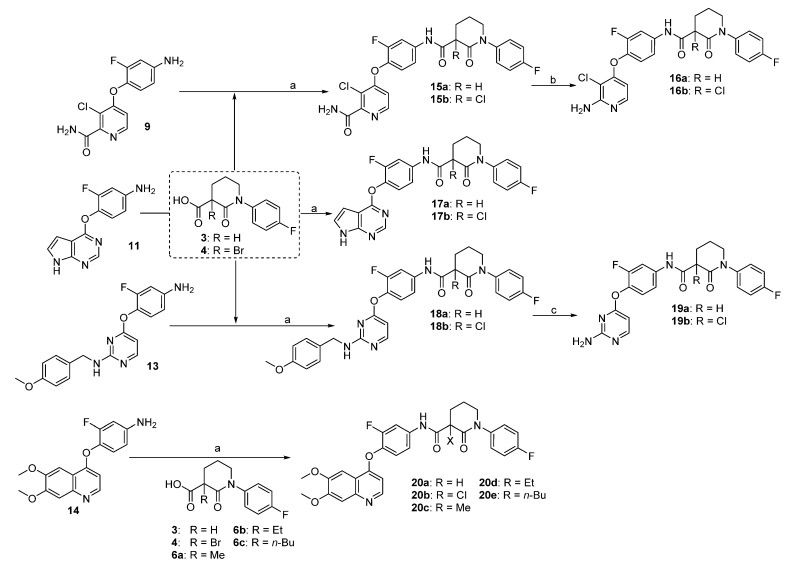
Synthesis of the newly designed c-Met inhibitors **15**–**20**.

Coupling of aromatic amines **9**, **11**, **13** or **14** with the 3-carboxypiperidin-2-one **3** in the presence of 1-ethyl-3-(3-dimethylaminopropyl)-carbodiimide hydrochloride (EDC-HCl) and *N*,*N*-dimethyl-4-amino pyridine (DMAP) gave the corresponding amides **15a**, **17a**, **18a** or **20a**, respectively (Scheme 3). When bromide **4** was used as the carboxylic acid component, halo-exchanged products **15b**, **17b**, **18b**, **20b** were observed (confirmed by NMR and MS). The aminopyridine-containing products **16a**–**b** were achieved after Hoffman degradation and the aminopyrimidine derivates **19a**–**b** were generated after treatment of **18a**–**b** with trifluoroacetic acid (TFA).

*Reagents and conditions*: (**a**) EDC-HCl, DMAP, 76% for **15a**, 68% for **15b**, 31% for **17a**, 42% for **17b**, 69% for **18a**, 57% for **18b**, 61% for **20a**, 54% for **20b**, 68% for **20c**, 58% for **20d**, 64% for **20e**; (**b**) PhI(OAc)_2_, 72% for **16a**, 76% for **16b**; (**c**) TFA, 71% for **19a**, 58% for **19b**.

### 2.2. Evaluation of Biological Activity

As illustrated in Table 1, all of the compounds bearing a 3-carboxypiperidin-2-one scaffold exhibit potent c-Met kinase inhibition activity. However, compounds lacking an α-substituent group (**15a**, **17a**, **18a**, **19a**, **20a**) only showed much less potent anti-c-Met kinase activity. When the α-proton was substituted by chlorine, the activity generally increased (*cf.* **15b** *vs.* **15a**, **16b** *vs.* **16a**, **20b** *vs.* **20a**). When alkyl groups (Me, Et, or *n*-Bu) were introduced to this position, the inhibitory effects were greatly enhanced (**20c**, **20d** and **20e** *vs.* **20a**). Among these three derivatives, the smallest methyl group was the most favorable among the compounds exerting inhibitory activity against c-Met kinase activity and c-Met-driven cell proliferation. Generally, 6,7-dimethoxyquinoline -containing analogues showed more potency than the pyrropyridine, pyrimidine, or aminopyrimidine counterparts (**20b** *vs.* **15b**, **16b**, **17b**, **18b**, **19b**) according to the biological activity results. The most potent analogue **20b** exhibited significant potency against c-Met kinase and c-Met-driven MKN45 cell proliferation, with IC_50_ values of 8.6 nM and 0.57 μM, respectively. Other three analogues **20c**–**e** with alkyl substitution in the piperidone moiety are also promising, showing inhibitory activity against c-Met enzymatic activities with the IC_50_s of 11.2~64.0 nM and inhibiting MKN45 cell proliferation with IC_50_s of 0.65~16.0 μM, individually.

**Table 1:** SAR of the compounds bearing a 3-carboxypiperidin-2-one scaffold. 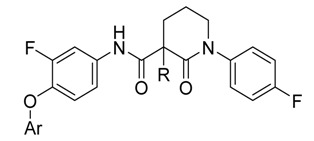

Cmpd	Ar	R	c-Met IC_50_ (nM)	MKN45 *^a^* IC_50_ (μM)	Compd	Ar	R	c-Met IC_50_ (nM)	MKN45 *^a^* IC_50_ (μM)
**15a**	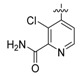	H	63.9%@10 μM	NT *^b^*	**19a**	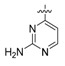	H	59.1%@10 μM	NT
								27.5%@1 μM	
**15b**	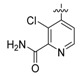	Cl	90.2%@10 μM	NT	**19b**	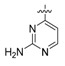	Cl	52.5%@10 μM	NT
			38.7%@1 μM					28.3%@1 μM	
**16a**	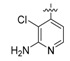	H	427.0 ± 6.1	NT	**20a**	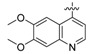	H	38.4%@10 μM	NT
**16b**	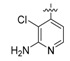	Cl	81.0 ± 7.6	NT	**20b**	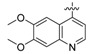	Cl	8.6 ± 1.6	0.57 ± 0.04
**17a**	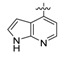	H	70.7%@10 μM	NT	**20c**	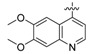	Me	11.2 ± 4.1	0.65 ± 0.13
			41.9%@1 μM						
**17b**	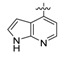	Cl	15.4%@10 μM	NT	**20d**	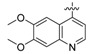	Et	19.1 ± 4.5	2.95 ± 0.12
**18a**	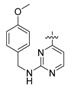	H	28.4%@10 μM	NT	**20e**	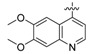	*^n^*Bu	64.0 ± 10.8	16.0 ± 0.8
**18b**	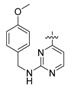	Cl	10%@10 μM	NT	BMS-777607	—	—	3.7 ± 1.3	0.29 ± 0.02

*^a^*: MKN45, human gastric cancer cell line that expresses elevated levels of constitutively active c-Met; *^b^*: NT, Not tested.

### 2.3. Molecular Modeling

**Figure 3:**
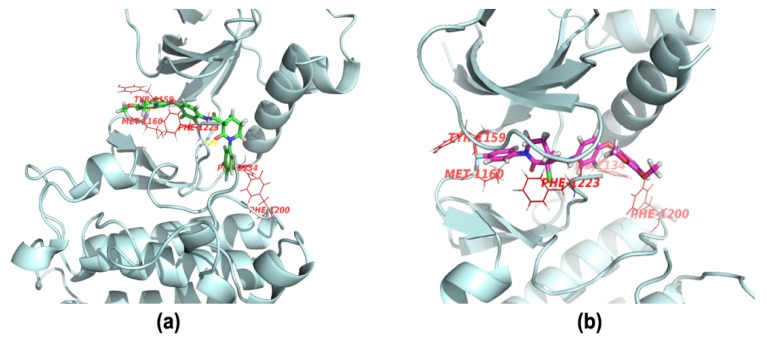
Binding poses of compounds (*R*)-**20b** and (*S*)-**20b** with c-Met.

To further elucidate the binding mode of compounds, docking analysis was performed. In our study, the co-crystal structure of BMS-777607 with c-Met kinase (PDB ID:3F82) was selected as the docking model. The inhibitor was docked using the GLIDE docking algorithm [27] in the XP (extra precision) mode. A binding model for (*R*)-**20b** in the ATP binding site is presented in Figure 3a. The resulting model successfully identifies key hydrogen bond interaction and hydrophobic interactions between the ligands and residues of the protein’s ATP binding pocket. The carbonyl oxygen of the 3-carboxypiperidin-2-one and the nitrogen atom of the quinoline ring in **20b** formed hydrogen bonding interactions with Asp1222 and Met1160, respectively. π-π Interactions were formed between the phenyl ring (moiety C) and Phe1223. In addition, hydrophobic interactions were formed between the phenyl ring (moiety A) in **20b** and Phe1134, Phe1200. A binding model for (*S*)-**20b** in the ATP binding site is presented in Figure 3b. However, this compound failed to dock into the binding pocket, as the orientation of the ligand in the binding model was opposite to that of BMS-777607. Therefore, we postulate that the requisite chirality for these compound may be the *R*-configuration. We are now seeking an efficient route to access the enantiomers, and the optical pure compounds will be synthesized and evaluated in the due course.

## 3. Experimental

### 3.1. General Information

All chemical reagents were used as supplied unless indicated. Solvents used in organic reactions were distilled under an inert atmosphere. Unless otherwise noted, all reactions were carried out at room temperature and performed under a positive pressure of argon. Flash column chromatography was performed on silica gel (200–300 mesh, Qingdao Haiyang Chemical Co., Ltd, Qingdao, China). Analytical thin layer chromatography (TLC) was performed on glass plates pre-coated with a 0.25 mm thickness of silica gel. ^1^H-NMR and ^13^C-NMR spectra were taken on a Jeol JNM-ECP 600 spectrometer (Jeol Ltd., Tokyo, Japan) at room temperature. Chemical shifts of the ^1^H-NMR spectra are expressed in ppm relative to the solvent residual signal 7.26 in CDCl_3_ or to tetramethylsilane (δ = 0.00). Chemical shifts of the ^13^C-NMR spectra are expressed in ppm relative to the solvent signal 77.00 in CDCl_3_ or to tetramethylsilane (δ = 0.00) unless otherwise noted. Electrospray (ESI) mass spectra were recorded on a Global Q-TOF mass spectrometer (Waters, Wilford, MA, USA).

### 3.2. Synthesis

*Isobutyl 1-(4-fluorophenyl)-2-oxopiperidine-3-carboxylate* (**2**). 1-Fluoro-4-iodobenzene (2.22 g, 10 mmol) and piperidin-2-one (1.2 g, 12 mmol) were added to dry DMF (30 mL), followed by the addition of K_3_PO_4_ (6.36 g, 30 mmol) and CuI (190 mg, 0.1 mmol). The mixture was heated to 100 °C for 12 h before filtering through Celite. After washing with ethyl acetate (3 × 10 mL), the combined organic phase was concentrated and the residue was purified by column chromatography to give 1-(4-fluorophenyl)piperidin-2-one (1.73 g, 90%) as a yellow solid. This *N*-arylpiperidin-2-one (386 mg, 2 mmol) was dissolved in dry THF (20 mL) and cooled to −78 °C. After the addition of *tert-*BuLi (1.4 mL, 1.6 M in THF, 2.2 mmol) and stirring at this temperature for 4 h, isobutyl chlorofomate (400 μL, 2 mmol) was added. Ten min later, the reaction was quenched by addition of saturated aq. NH_4_Cl (2 mL). The mixture was diluted with water (20 mL) and extracted with EtOAc (3 × 20 mL). The combined organic layer was washed with brine, dried over Na_2_SO_4_, and concentrated *in vacuo*. The residue was purified by column chromatography to give compound **2** (480 mg, 82%) as a yellow wax. ^1^H-NMR (600 MHz, CDCl_3_) δ 7.25–7.20 (m, 2H, ArH), 7.09–7.03 (m, 2H，ArH), 3.99 (dd, 1H, *J* = 10.6, 6.7 Hz, CH), 3.83 (d, 1 H, *J* = 6.7 Hz, CHH), 3.70–3.61 (m, 1H, CHH), 3.58 (t, 1 H, *J* = 6.9 Hz, CH), 2.32–2.24 (m, 1H, CHH), 2.23–2.16 (m, 1H, CHH), 2.12–2.04 (m, 1H, CHH), 2.02–1.87 (m, 2H, CH, CHH), 0.94 (d, 6 H, *J* = 6.6 Hz, CH_3_); ^13^C-NMR (150 MHz, CDCl_3_) δ 171.0, 166.3, 162.1, 160.4, 138.8, 127.9, 116.2, 100.0, 71.5, 51.6, 49.6, 27.8, 25.3, 21.4, 19.1; HR-MS (ESI) Calcd for C_16_H_21_FNO_3_ [M + H]^+^ 294.1506, Found 294.1518.

*1-(4-Fluorophenyl)-2-oxopiperidine-3-carboxylic acid* (**3**). To a solution of **2** (217 mg, 0.74 mmol) in THF/MeOH/H_2_O (1/1/1, 3 mL in total) at 0 °C was added LiOH monohydrate (94 mg, 2.2 mmol). The reaction mixture was warmed to room temperature and stirred for 5 h. The solution was acidified to pH 1 with 1 mol/L HCl and extracted with EtOAc (3 × 20 mL). The organic extracts were combined and washed with brine (2 × 5 mL). Evaporation of the solvent gave the corresponding acid **3** (152 mg, 87%) as a white solid. ^1^H-NMR (600 MHz, CDCl_3_) δ 7.33–7.28 (m, 1H, ArH), 7.25–7.19 (m, 1H, ArH), 3.69–3.55 (m, 2H, NCH2), 3.43 (dd, 1H, *J* = 8.2, 6.5 Hz, CH), 2.16–2.10 (m, 1 H, CHH), 2.08–2.02 (m, 1H, CHH), 1.98–1.91 (m, 1H, CHH), 1.91–1.83 (m, 1H, CHH); ^13^C-NMR (150 MHz, CDCl_3_) δ 174.3, 170.2, 161.3, 159.6, 138.7, 127.9, 115.6, 51.8, 50.3, 27.5, 21.6; HR-MS (ESI) Calcd for C_12_H_13_FNO_3_ 238.0880 [M + H]^+^, found 238.0910.

*3-Bromo-1-(4-fluorophenyl)-2-oxopiperidine-3-carboxylic acid* (**4**). To a solution of acid **3** (220 mg, 0.93 mmol) in Et_2_O (5 mL) was added liquid Br_2_ (48 μL, 0.93 mmol) at 0 °C. The reaction mixture was stirred for 2 h before concentrated *in vacuo*. The residue was purified by column chromatography, giving compound **4** (265 mg, 91%) as white solid. ^1^H-NMR (600 MHz, acetone-*d_6_*) δ 13.03 (s, 1H, OH), 7.44–7.40 (m, 2H, ArH), 7.25–7.20 (m, 2H, ArH), 4.04 (td, 1H, *J* = 12.1, 4.6 Hz, NCHH), 3.82 (ddt, 1H, *J* = 13.0, 6.3, 2.4 Hz, NCHH), 2.77–2.69 (m, 1H, CH*H*), 2.62–2.56 (m, 1H, CH*H*), 2.53–2.43 (m, 1H, CHH), 2.19–2.12 (m, 1H, CHH); ^13^C-NMR (150 MHz, acetone-*d_6_*) δ 166.4, 162.5, 160.9, 140.6, 140.6, 129.0, 129.0, 116.2, 52.1, 32.3, 20.4.

#### 3.2.1. General Procedure for the Synthesis of Isobutyl 1-(4-Fluorophenyl)-3-alkyl-2-oxopiperidine-3-carboxylates **5a**–**c**

To a solution of compound **2** (586 mg, 2 mmol) in dry THF (10 mL) at 0 °C was added NaH (72 mg, 80% suspension in mineral oil, 2.4 mmol) in portions. Thirty min later, alkyl halide (MeI, EtBr, or *n*-BuBr, 2.6 mmol) was added slowly and the reaction mixture was stirred at this temperature for another 5 h. When TLC showed all the starting material consumed, the reaction mixture was quenched with 0.5 mol/L HCl, diluted with water (20 mL) and extracted with EtOAc (3 × 20 mL). The combined organic layer was washed with brine, dried over Na_2_SO_4_ and concentrated. The residue was purified by column chromatography to give the desired compound as pale yellow oil.

*Isobutyl 1-(4-fluorophenyl)-3-methyl-2-oxopiperidine-3-carboxylate* (**5a**). 87% yield; ^1^H-NMR (600 MHz, CDCl_3_) δ 7.25–7.19 (m, 1 H, ArH), 7.12–7.01 (m, 1H, ArH), 4.06–3.88 (m, 2H, OCH_2_), 3.78–3.58 (m, 1H, CH*H*), 2.47–2.31 (m, 1H, CH*H*), 2.10–1.94 (m, 3H, CH_2, _CH), 1.94–1.84 (m, 1H, CH*H*), 1.57 (d, 3H, *J* = 2.4 Hz, CH_3_), 0.97 (d, 3H, *J* = 2.0 HzCH_3_), 0.96 (d, 3H, *J* = 2.1 Hz, CH_3_); ^13^C-NMR (150 MHz, CDCl_3_) δ 173.8, 170.1, 161.9, 160.2, 139.3, 127.8, 116.0, 115.9, 71.5, 51.9, 51.3, 33.6, 27.8, 22.9, 20.4, 19.2; HR-MS (ESI) Calcd for C_17_H_23_FNO_3_ 308.1662 [M + H]^+^, found 308.1599.

*Isobutyl 3-ethyl-1-(4-fluorophenyl)-2-oxopiperidine-3-carboxylate* (**5b**). 76% yield; ^1^H-NMR (600 MHz, CDCl_3_) δ 7.24–7.18 (m, 2H, ArH), 7.08–7.03 (m, 2H, ArH), 4.01–3.90 (m, 2H, OCH_2_), 3.72–3.65 (m, 1 H, CHH), 3.63–3.56 (m, 1H, CHH), 2.31–2.24 (m, 1H, CHH), 2.16–2.09 (m, 1H, CHH), 2.10–2.04 (m, 1H, CHH), 2.02–1.91 (m, 4H, CH_2_), 0.98 (t, 3H, *J* = 7.4 Hz, CH_3_), 0.96 (d, 6H, *J* = 2.1 Hz, 2 × CH_3_); ^13^C-NMR (150 MHz, CDCl_3_) δ 173.6, 170.0, 161.8, 160.2, 139.3, 127.8, 116.0, 71.5, 51.9, 51.3, 33.6, 30.1, 27.8, 22.9, 20.4, 19.8; HR-MS (ESI) Calcd for C_18_H_25_FNO_3_ 322.1819 [M + H]^+^, found 322.1830.

*Isobutyl 3-butyl-1-(4-fluorophenyl)-2-oxopiperidine-3-carboxylate* (**5c**). 83% yield; ^1^H-NMR (600 MHz, CDCl_3_) δ 7.23–7.18 (m, 2H, ArH), 7.08–7.02 (m, 2H, ArH), 4.00–3.89 (m, 2H, OCH_2_), 3.72–3.66 (m, 1H, CHH), 3.61–3.56 (m, 1H, CHH), 2.32–2.26 (m, 1H, CHH), 2.10–1.86 (m, 6H), 1.46–1.38 (m, 1H, CHH), 1.37–1.29 (m, 2H, CH_2_), 1.30–1.21 (m, 1H, CHH), 0.96 (d, 6H, *J* = 2.1 Hz, 2 × CH_3_), 0.90 (t, 3H, *J* = 7.2 Hz, CH_3_); ^13^C-NMR (150 MHz, CDCl_3_) δ 173.5, 169.4, 161.8, 160.2, 139.4, 127.8, 115.9, 71.5, 54.9, 51.6, 35.7, 30.1, 27.0, 23.2, 20.7, 19.2, 14.1; HR-MS (ESI) Calcd for C_20_H_29_FNO_3_ 350.2132, [M + H]^+^, found 350.2122.

#### 3.2.2. 1-(4-Fluorophenyl)-3-alkyl-2-oxopiperidine-3-carboxylic Acids **6a**–**c** were Prepared by a Procedure Similar to that of Compound **3**.

*3-Methyl-1-(4-fluorophenyl)-2-oxopiperidine-3-carboxylic acid* (**6a**). White solid; 88% yield; ^1^H-NMR (600 MHz, DMSO-*d_6_*) δ 12.59 (s, 1 H, OH), 7.31–7.25 (m, 2H, ArH), 7.24–7.17 (m, 2H, ArH), 3.65 (dt, 1H, *J* = 12.1, 6.1 Hz, NCHH), 3.59 (dt, 1H, *J* = 11.9, 5.8 Hz, NC*H*H), 2.25–2.18 (m, 1H, CHH), 1.95–1.87 (m, 2H, CH_2_), 1.87–1.80 (m, 1H, CHH), 1.37 (s, 3H, CH_3_); ^13^C-NMR (150 MHz, DMSO-*d_6_*) δ 174.9, 169.7, 160.9, 159.3, 139.8, 128.2, 115.6, 51.2, 50.4, 32.8, 22.6, 19.8; HR-MS (ESI) Calcd for C_13_H_15_FNO_3_ 252.1036, [M + H]^+^, found 252.1040.

*3-Ethyl-1-(4-fluorophenyl)-2-oxopiperidine-3-carboxylic acid* (**6b**). white solid; 79% yield; ^1^H-NMR (600 MHz, DMSO-*d_6_*) δ 12.79 (s, 1 H, OH), 7.26–7.23 (m, 2H, ArH), 7.16–7.12 (m, 2H, ArH), 3.92 (dt, *J* = 12.7, 6.5 Hz, 1H, NCHH), 3.74 (dt, *J* = 12.6, 6.4 Hz, 1H, NCHH), 2.56–2.50 (m, 1H, CHH), 2.20 (q, *J* = 6.7 Hz, 1H, CHH), 2.05–2.01 (m, 1H, CHH), 2.01–1.91 (m, 2H, CHH), 1.84 (dt, *J* = 12.3, 7.0 Hz, 1 H, CHH), 0.71 (t, *J* = 6.6 Hz, 3H, CH_3_); ^13^C-NMR (150 MHz, DMSO-*d_6_*) δ 174.8, 169.8, 161.2, 159.6, 139.5, 128.4, 115.8, 51.7, 50.9, 33.6, 29.7, 22.6, 18.8; HR-MS (ESI) Calcd for C_14_H_17_FNO_3_ 266.1193, [M + H]^+^, found 266.1201.

*3-Butyl-1-(4-fluorophenyl)-2-oxopiperidine-3-carboxylic acid* (**6c**). White solid; 84% yield; ^1^H-NMR (600 MHz, DMSO-*d_6_*) δ 12.66 (s, 1H, OH), 7.25–7.20 (m, 2H, ArH), 7.14 (dd, 2H, *J* = 8.6, 7.0 Hz, ArH), 3.96–3.87 (m, 1H, CHH), 3.78–3.72 (m, 1H, CHH), 2.49–2.42 (m, 1H, CHH), 2.22 (dt, *J* = 19.0, 7.5 Hz, 2H, CH_2_), 2.03–1.91 (m, 3H, CHH*,* CH_2_), 1.38–1.21 (m, 4H, CH_2_CH_2_), 0.88 (t, 3 H, *J* = 6.4 Hz, CH_3_); ^13^C-NMR (150 MHz, DMSO-*d_6_*) δ 174.7, 169.5, 161.6, 160.0, 139.6, 127.9, 115.6, 54.6, 51.3, 35.4, 30.3, 23.2, 20.7, 14.2; HR-MS (ESI) Calcd for C_16_H_21_FNO_3_ 294.1506, [M + H]^+^, found 294.1496.

*3,4-Dichloropicolinamide* (**8**). To a solution of 2,2,6,6-tetramethylpiperidine (1.56 g, 11 mmol) in dry ether (20 mL) at 0 °C was added *n*-BuLi (4.4 mL, 2.5 M in THF, 11 mmol) slowly. The reaction mixture was stirred at this temperature for 30 min before cooled to −78 °C. A solution of 3,4-dichloropyridine (1.48 g, 10 mmol) in dry ether (5 mL) was injected via syringe to the above reaction mixture and stirred for 2 h before trimethylsilyl isothiocyanate (15 mmol) was added. After warmed to room temperature, the reaction was quenched by the addition of HOAc (2 mL) and water (10 mL), and then let to stir overnight. The suspension was filtered and washed with cold water, giving the title compound as a gray solid (686 mg, 40%). ^1^H-NMR (DMSO-*d_6_*, 600 MHz) δ 8.50 (d, 1H, *J* = 5.2 Hz, ArH), 8.12 (br s, 1H, CONH_2_), 7.83 (d, 1H, *J* = 5.2 Hz, ArH), 7.82 (br s, 1H, CONH_2_).

*4-(4-Amino-2-fluorophenoxy)-3-chloropicolinamide* (**9**). To a solution of 4-amino-2-fluorophenol (465 mg, 3.65 mmol) in DMF (10 mL) was added potassium *tert*-butoxide (440 mg, 3.95 mmol). Thirty min later, 3,4-dichloropicolinamide (**8**) was added and the solution was heated to 50 °C. When TLC showed all the starting materials consumed, the reaction mixture was diluted with EtOAc (50 mL), washed with saturated NaHCO_3_, brine, and dried over Na_2_SO_4_. After concentration, the residue was purified by column chromatography giving the title compound as a pale yellow solid (580 mg, 79%). ^1^H-NMR (DMSO-*d_6_*, 600 MHz) δ 8.29 (d, 1 H, *J* = 5.6 Hz, ArH), 7.00 (t, 1H, *J* = 8.8 Hz, ArH), 6.79 (d, 1H, *J* = 5.6 Hz, ArH), 6.63–6.55 (m, 2H, ArH); ^13^C-NMR (DMSO-*d_6_*, 150 MHz) δ 166.6, 160.8, 154.1, 153.9, 149.0, 148.7, 128.5, 123.7, 115.9, 110.1, 110.0, 101.3.

*4-((7H-Pyrrolo[2,3-d]pyrimidin-4-yl)oxy)-3-fluoroaniline* (**11**). The solution of 4-chloro-*7H*-pyrrolo-[2,3-d]pyrimidine (1.0 g, 6.5 mmol) and 2-fluoro-4-nitrophenol (1.5 g, 9.5 mmol) in bromobenzene (5 mL) was heated at 130 °C for 4 h in a sealed tube. After that, the reaction mixture was cooled to room temperature, diluted with Et_2_O (5 mL) and filtered. Recrystallization in MeOH gave 4-(2-fluoro-4-nitrophenoxy)-7*H*-pyrrolo[2,3-*d*]pyrimidine as a yellow solid (1.6 g, 85%), which was used for the next step directly. To a solution of this nitro compound in THF (5 mL) and MeOH (5 mL) was added zinc powder (130 mg, 2 mmol) and NH_4_Cl (270 mg, 5 mmol). The reaction mixture was stirred at room temperature for 5 h before filtered through a Celite pad. The filtrate was diluted with EtOAc, washed with water, dried over Na_2_SO_4_ and concentrated. The residue was purified by column chromatography giving compound **11** (115 mg, 69%) as a brown solid. ^1^H-NMR (600 MHz, DMSO-*d_6_*_)_ δ 12.19 (s, 1H, NH), 8.28 (s, 1H, CH), 7.44 (t, 1H, *J* = 3.1 Hz, ArH), 7.00 (t, 1H, *J* = 8.9 Hz, ArH), 6.48 (dd, 1H, *J* = 13.0, 2.6 Hz, ArH), 6.45 (dd, 1H, *J* = 3.4, 1.5 Hz, CH), 6.42–6.39 (m, 1H, CH), 5.35 (s, 2H, NH_2_).

*2-Chloro-4-(2-fluoro-4-nitrophenoxy)pyrimidine* (**12**). 2-Fluoro-4-nitrophenol (314 mg, 2 mmol), K_2_CO_3_ (304 mg, 2.2 mmoo) and 2,4-dichloropyrimidine (300 mg, 2 mmol) were dissolved in DMF (20 mL) and heated at 100 °C for 2 h. the reaction mixture was concentrated, diluted with EtOAc (100 mL), washed with water, brine, and concentrated *in vacuo*. The residue was purified by column chromatography giving compound **12** (324 mg, 65%) as a white solid. ^1^H-NMR (600 MHz, DMSO-*d_6_*) δ 8.78 (d, *J* = 5.7 Hz, 1H, ArH), 8.44 (dd, 1H *J* = 10.2, 2.7 Hz, ArH), 8.25 (ddd, 1H, *J* = 9.0, 2.7, 1.3 Hz, ArH), 7.82 (dd, 1H, *J* = 9.0, 7.7 Hz, ArH), 7.49 (d, 1H, *J* = 5.7 Hz, ArH).

*4-(4-Amino-2-fluorophenoxy)-N-(4-methoxybenzyl)pyrimidin-2-amine* (**13**). To the solution of compound **12** (239 mg 1mmol) and 4-methoxybenzylamine (192 mg, 1.4 mmol) in DMF (8 mL) was added K_2_CO_3_ (152 mg, 1.1 mmol). The reaction mixture was heated at 100 °C for 1 h before concentrated in vacuo. After diluted with EtOAc, the solution was washed with water and brine, and then concentrated. The residue was purified by column chromatography giving a yellow solid (231 mg, 68%), which was treated by zinc powder and NH_4_Cl as described for the preparation of compound **11**. After workup and purification, compound **13** was obtained as a brown solid (115 mg, 69%). ^1^H-NMR (600 MHz, CDCl_3_) δ 8.06 (br s, 1H, ArH), 7.12 (br s, 2H, ArH), 6.92 (t, 1H, *J* = 8.6 Hz, ArH), 6.80 (d, 2H, *J* = 8.2 Hz, ArH), 6.47 (dd, 1H, *J* = 11.8, 2.7 Hz, ArH), 6.45–6.39 (m, 1H, ArH), 6.15 (d, 1H, *J* = 5.8 Hz, ArH), 4.36 (br s, 2H, NH_2_), 3.92 (br s, 2H, CH_2_), 3.78 (s, 3H, CH_3_).

*4-((6,7-Dimethoxyquinolin-4-yl)oxy)-3-fluoroaniline* (**14**). The procedure used was similar to that used for the synthesis of compound **11**. Compound **14** was obtained as a brown solid in 76% yield. ^1^H-NMR (600 MHz, CDCl_3_) δ 8.47 (d, 1H, *J* = 5.1 Hz, ArH), 7.58 (s, 1H, ArH), 7.40 (s, 1H, ArH), 7.02 (t, 1H, *J* = 8.6 Hz, ArH), 6.55 (dd, 1H, *J* = 12.0, 2.7 Hz, ArH), 6.49 (dd, 1H, *J* = 8.9, 2.6 Hz, ArH), 6.40 (d, 1H, *J* = 5.0 Hz, ArH), 4.05 (s, 3H, OCH_3_), 4.03 (s, 3H, OCH_3_), 3.84 (br s, 2H, NH_2_).

#### 3.2.3. General Procedure for the Preparation of **15a**–**b**, **17a**–**b**, **18a**–**b** and **20a**–**b**

EDC-HCl (1.2 g, 6.25 mmol) was added to a suspension of the carboxylic acid (2.5 mol of **3**, **4**, or **6a**–**c**) and the amine (2.5 mmol of **9**, **11**, **13** or **14**) in THF (25 mL) at 0 °C followed by DMAP (30 mg, 0.25 mmol). The reaction mixture was warmed to room temperature and stirred overnight. After diluted with EtOAc (150 mL), the whole mixture was washed with 1 M HCl (3 × 10 mL), 5% NaHCO_3_ (3 × 10 mL), and brine (3 × 10 mL), dried over Na_2_SO_4_, and concentrated *in vacuo*. The residue was purified by column chromatography to give corresponding amide.

*3-Chloro-4-(2-fluoro-4-(1-(4-fluorophenyl)-2-oxopiperidine-3-carboxamido)phenoxy)picolinamide* (**15a**, from **9** and **3**): 76% yield; ^1^H-NMR (600 MHz, DMSO-*d_6_*) δ 10.56 (s, 1H, NH), 8.33 (d, 1H, *J* = 5.5 Hz, ArH), 8.07 (s, 1H, NH), 7.91 (dd, 1H, *J* = 12.9, 2.4 Hz, ArH), 7.77 (s, 1H, NH), 7.44 (dd, 1H, *J* = 8.9, 2.4 Hz, ArH), 7.41 (t, 1H, *J* = 8.8 Hz, ArH), 7.36–7.32 (m, 2H, ArH), 7.26–7.20 (m, 2H, ArH), 6.84 (dd, 1H, *J* = 5.5, 1.1 Hz, ArH), 3.76–3.68 (m, 1H, CH), 3.65–3.57 (m, 2H, CH_2_), 2.21–2.13 (m, 2H, CH_2_), 2.12–2.04 (m, 1H, CHH), 1.96–1.87 (m, 1H, CHH); ^13^C-NMR (150 MHz, DMSO-*d_6_*) δ 169.3, 166.5, 160.9, 160.0, 159.3, 154.2, 153.8, 152.2, 148.7, 139.4, 138.5, 138.4, 134.8, 134.7, 128.3, 128.2, 123.7, 116.3, 115.9, 115.6, 115.4, 110.6, 107.9, 107.8, 54.9, 51.2, 50.5, 48.6, 24.8, 21.2; MS (ESI pos ion) *m/z*: calcd for C_24_H_19_ClF_2_N_4_O_4_ 500.1, found 501.1 [M + H]^+^; HR-MS (ESI) Calcd for C_24_H_20_ClF_2_N_4_O_4_ 501.1141 [M + H]^+^, found 501.1160.

*3-Chloro-4-(4-(3-chloro-1-(4-fluorophenyl)-2-oxopiperidine-3-carboxamido)-2-fluorophenoxy)-picolinamide* (**15b**, from **9** and **4**) 68% yield; ^1^H-NMR (600 MHz, CDCl_3_) δ 10.11 (s, 1H, NH), 8.24 (d, 1H, *J* = 5.5 Hz, ArH), 7.80 (dd, 1H, *J* = 11.9, 2.5 Hz, ArH), 7.54 (d, 1H, *J* = 3.9 Hz, ArH), 7.33–7.22 (m, 3H, ArH), 7.18–7.11 (m, 3H, NH), 6.68 (dd, 1H, *J* = 5.5, 1.1 Hz, ArH), 6.15 (d, 1H, *J* = 3.4 Hz, ArH), 3.85–3.78 (m, 1H, CHH), 3.73–3.68 (m, 1H, CHH), 2.96–2.87 (m, 1H, CHH), 2.65–2.56 (m, 1H, CHH), 2.45–2.34 (m, 1H, CHH), 2.17–2.07 (m, 1H, CHH);^ 13^C-NMR (150 MHz, CDCl_3_) δ 166.8, 166.0, 164.9, 162.6, 161.8, 160.9, 154.7, 153.0, 148.3, 147.0, 137.9, 136.8, 136.8, 136.8, 127.9, 127.8, 123.4, 121.2, 116.7, 116.6, 116.5, 111.7, 109.8, 109.6, 64.4, 52.6, 33.8, 19.4; MS (ESI pos ion) *m/z*: calcd for C_24_H_18_Cl_2_F_2_N_4_O_4_ 534.1 found 535.1 [M + H]^+^; HR-MS (ESI) Calcd for C_24_H_19_Cl_2_F_2_N_4_O_4_ 535.0752 [M + H]^+^, found 535.0764.

*N-(4-((7H-pyrrolo[2,3-d]pyrimidin-4-yl)oxy)-3-fluorophenyl)-1-(4-fluorophenyl)-2-oxopiperidine-3-carboxamide* (**17a**, from **11** and **3**): 31% yield, ^1^H-NMR (600 MHz, CDCl_3_) δ 11.02 (s, 1H, NH), 10.19 (s, 1H, NH), 8.87 (s, 1H, ArH), 8.54 (s, 1H, ArH), 8.13–7.93 (m, 1H, ArH), 7.81 (d, 1H, *J* = 11.6 Hz, ArH), 7.42 (s, 1H, ArH), 7.31–7.20 (m, 2H, ArH), 7.16–7.03 (m, 2H, ArH), 6.92 (s, 1H, ArH), 3.74–3.59 (m, 2H, NCH_2_), 3.59–3.47 (m, 1H, CH), 2.63–2.51 (m, 1H, CHH), 2.26–2.16 (m, 1 H, CH*H*), 2.15–2.07 (m, 1 H, CHH), 2.08–1.98 (m, 1 H, CHH); ^13^C-NMR (150 MHz, CDCl_3_) δ 169.5, 168.3, 162.5, 160.9, 156.2, 155.9, 154.6, 152.4, 151.1, 139.8, 138.5, 134.5, 134.2, 128.2, 128.1, 126.2, 123.0, 116.9, 116.8, 116.7, 116.6, 115.8, 115.6, 109.4, 109.2, 100.3, 53.1, 47.8, 47.7, 29.7, 22.8, 21.6; MS (ESI pos ion) *m/z*: calcd for C_24_H_19_F_2_N_5_O_3_ 463.1, found 464.2 [M + H]^+^; HR-MS (ESI) Calcd for C_24_H_20_F_2_N_5_O_3_ 464.1534 [M + H]^+^, found 464.1547.

*N-(4-((7H-pyrrolo[2,3-d]pyrimidin-4-yl)oxy)-3-fluorophenyl)-3-chloro-1-(4-fluorophenyl)-2-oxo-piperidine-3-carboxamide* (**17b**, from **11** and **4**): 42% yield; ^1^H-NMR (600 MHz, CDCl_3_) δ 8.25 (s, 1H, ArH), 7.89 (dd, *J* = 12.5, 2.5 Hz, 1H, ArH), 7.50 (d, *J* = 3.6 Hz, 1H, ArH), 7.48 (dd, *J* = 2.5, 1.3 Hz, 1H, ArH), 7.46–7.42 (m, 3H, ArH), 7.38 (t, *J* = 8.7 Hz, 1H, ArH), 7.25–7.19 (m, 2H, ArH), 6.64 (d, *J* = 3.6 Hz, 1H, ArH), 3.98–3.88 (m, 1H. CHH), 3.84–3.78 (m, 1H. CHH), 2.96 (ddd, *J* = 14.8, 11.6, 3.2 Hz, 1H. CHH), 2.59–2.52 (m, 1H. CHH), 2.40–2.31 (m, 1H. CHH), 2.21–2.11 (m, 1H. CHH); ^13^C-NMR (150 MHz, CDCl_3_) δ 169.7, 168.5, 162.7, 160.9, 156.3, 155.9, 154.7, 152.4, 151.3, 139.8, 138.6, 134.7, 134.5, 128.4, 128.3, 126.4, 123.6, 117.0, 116.8, 116.7, 116.6, 115.9, 115.7, 109.6, 109.4, 100.6, 64.5, 53.5, 47.9, 47.7, 29.9, 22.8, 21.8; MS (ESI pos ion) *m/z*: calcd for C_24_H_18_ClF_2_N_5_O_3_ 497.1, found 498.0 [M + H]^+^; HR-MS (ESI) Calcd for C_24_H_19_ClF_2_N_5_O_3_ 498.1145 [M + H]^+^, found 498.1155.

*N-(3-fluoro-4-((2-((4-methoxybenzyl)amino)pyrimidin-4-yl)oxy)phenyl)-1-(4-fluorophenyl)-2-oxo-piperidine-3-carboxamide* (**18a**, from **13** and **3**): 69% yield, ^1^H-NMR (600 MHz, CDCl_3_) δ 10.19 (s, 1H, NH), 8.06 (s, 1H, ArH), 7.75 (d, 1H, *J* = 12.2 Hz, ArH), 7.24–7.18 (m, 3H, ArH), 7.11 (t, 2H, *J* = 8.3 Hz, ArH), 7.06 (t, 1H, *J* = 8.4 Hz, ArH), 7.00 (d, 1H, *J* = 8.6 Hz, ArH), 6.78 (s, 2H, ArH), 6.23 (s, 1H, ArH), 4.24 (s, 2H, CH_2_), 3.76 (s, 3H, OCH_3_), 3.71–3.62 (m, 2H, NCH_2_), 3.62–3.57 (m, 1H, CH), 2.58–2.48 (m, 1H, CHH), 2.25–2.14 (m, 1H, CHH), 2.13–2.05 (m, 1H, CHH), 2.05–1.97 (m, 1H, CHH); ^13^C-NMR (150 MHz, CDCl_3_) δ 169.3, 166.0, 162.4, 160.8, 159.0, 156.7, 154.8, 138.4, 129.3, 128.2, 128.1, 123.7, 116.6, 116.5, 115.3, 114.0, 55.4, 52.7, 47.7, 22.9, 21.7; MS (ESI pos ion) *m/z*: calcd for C_30_H_27_F_2_N_5_O_4_ 559.2, found 560.2 [M + H]^+^; HR-MS (ESI) Calcd for C_30_H_28_F_2_N_5_O_4_ 560.2109 [M + H]^+^, found 560.2125.

*3-Chloro-N-(3-fluoro-4-((2-((4-methoxybenzyl)amino)pyrimidin-4-yl)oxy)phenyl)-1-(4-fluorophenyl)-2-oxopiperidine-3-carboxamide* (**18b**, from **13** and **4**):57% yield; ^1^H-NMR (600 MHz, CDCl_3_) δ 9.96 (s, 1H, NH), 8.12 (s, 1H, ArH), 7.69 (dd, *J* = 11.8, 2.5 Hz, 1H, ArH), 7.25–7.22 (m, 2H, ArH), 7.19 (dd, 1H, *J* = 8.9, 2.4 Hz, ArH), 7.15–7.08 (m, 4H, ArH), 6.80 (d, 2H, *J* = 8.0 Hz, ArH), 6.18 (d, 1H, *J* = 5.7 Hz, ArH), 4.32 (s, 2H, CH_2_), 3.81–3.75 (m, 1H, CHH), 3.77 (s, 3H, OCH_3_), 3.69 (dt, 1H, *J* = 12.4, 4.9 Hz, CHH), 2.90 (ddd, 1H, *J* = 14.6, 11.3, 3.0 Hz, CHH), 2.63–2.54 (m, 1H, CHH), 2.41–2.31 (m, 1H, CHH), 2.15–2.06 (m, 1H, CHH); ^13^C-NMR (150 MHz, CDCl_3_) δ 166.9, 164.6, 162.6, 162.1, 160.9, 159.5, 158.9, 157.7, 155.3, 153.70, 138.0, 136.4, 136.3, 135.8, 135.8, 130.9, 129.1, 127.9, 127.8, 124.2, 116.7, 116.5, 115.7, 113.9, 64.4, 55.4, 55.3, 52.7, 45.0, 33.9, 19.5; MS (ESI pos ion) m/z: calcd for C_30_H_26_ClF_2_N_5_O_4_ 593.2, found 594.2 [M + H]^+^; HR-MS (ESI) Calcd for C_30_H_27_ClF_2_N_5_O_4_ 594.1720 [M + H]^+^, found 594.1733.

*N-(4-((6,7-dimethoxyquinolin-4-yl)oxy)-3-fluorophenyl)-1-(4-fluorophenyl)-2-oxopiperidine-3-carbox-amide* (**20a**, from **14** and **3**): 61% yield; ^1^H-NMR (600 MHz, CDCl_3_) δ 10.30 (s, 1H, NH), 8.51 (s, 1 H, ArH), 7.84 (dd, 1H, *J* = 12.1, 2.5 Hz, ArH), 7.69 (s, 1H, ArH), 7.59 (s, 1H, ArH), 7.29–7.26 (m, 1H, ArH), 7.25–7.21 (m, 2H, ArH), 7.18 (t, 1H, *J* = 8.6 Hz, ArH), 7.12 (t, 2H, *J* = 8.5 Hz, ArH), 6.49 (d, 1H, *J* = 5.5 Hz, ArH), 4.08 (s, 3H, OCH_3_), 4.06 (s, 3H, OCH_3_), 3.75–3.60 (m, 2H, CHH), 3.58–3.49 (m, 1H, CH), 2.60–2.47 (m, 1H, CHH), 2.29–2.19 (m, 1H, CHH), 2.14–1.97 (m, 2H, CH_2_); ^13^C-NMR (150 MHz, CDCl_3_) δ 169.3, 166.4, 154.6, 150.6, 145.7, 138.4, 128.1, 123.5, 116.6, 116.5, 116.2, 99.7, 56.7, 56.4, 52.7, 47.8, 29.8, 23.0, 22.8, 21.7; MS (ESI pos ion) *m/z*: calcd for C_29_H_25_F_2_N_3_O_5_ 533.2, found 534.2 [M + H]^+^; HR-MS (ESI) Calcd for C_29_H_26_F_2_N_3_O_5_ 534.1841 [M + H]^+^, found 534.1850.

*3-Chloro-N-(4-((6,7-dimethoxyquinolin-4-yl)oxy)-3-fluorophenyl)-1-(4-fluorophenyl)-2-oxopiperidine-3-carboxamide* (**20b**, from **14** and **4**): 54% yield; ^1^H-NMR (600 MHz, CDCl_3_) δ 10.05 (s, 1H, NH), 8.48 (d, 1H, *J* = 5.4 Hz, ArH), 7.79 (dd, 1H, *J* = 12.0, 2.5 Hz, ArH), 7.57 (s, 1H, ArH), 7.44 (s, 1H, ArH), 7.29–7.23 (m, 4H, ArH), 7.20 (t, 1H, *J* = 8.6 Hz, ArH), 7.13 (t, 2H, *J* = 8.4 Hz, ArH), 6.39 (d, 1H, *J* = 5.3 Hz, ArH), 4.05 (s, 3H, OCH_3_), 4.04 (s, 3H, OCH_3_), 3.80 (ddd, 1H, *J* = 14.5, 10.3, 4.7 Hz, CH*H*), 3.74–3.66 (m, 1H, CHH), 2.97–2.83 (m, 1H, CHH), 2.67–2.56 (m, 1H, CHH), 2.45–2.34 (m, 1H, CHH), 2.15–2.07 (m, 1H, CHH); ^13^C-NMR (150 MHz, CDCl_3_) δ 166.9, 164.8, 162.6, 161.0, 160.2, 155.3, 153.6, 153.1, 149.8, 148.6, 146.7, 137.9, 137.7, 137.6, 136.3, 136.2, 127.9, 127.8, 123.8, 116.6, 116.5, 116.4, 115.6, 113.0, 109.8, 109.7, 109.6, 109.6, 107.8, 107.74, 99.5, 99.5, 64.3, 56.3, 56.2, 52.7, 33.8, 29.8, 19.5; MS (ESI pos ion) *m/z*: calcd for C_29_H_24_ClF_2_N_3_O_5_ 567.1, found 568.1 [M + H]^+^; HR-MS (ESI) Calcd for C_29_H_25_ClF_2_N_3_O_5_ 568.1451 [M + H]^+^, found 568.1461.

*N-(4-((6,7-dimethoxyquinolin-4-yl)oxy)-3-fluorophenyl)-1-(4-fluorophenyl)-3-methyl-2-oxopiperidine-3-carboxamide* (**20c**, from **14** and **6a**): 68% yield; ^1^H-NMR (600 MHz, CDCl_3_) δ 10.01 (s, 1H, NH), 8.47 (d, 1H, *J* = 5.3 Hz, ArH), 7.82 (dd, 1H, *J* = 12.2, 2.5 Hz, ArH), 7.58 (s, 1H, ArH), 7.42 (s, 1H, ArH), 7.25–7.20 (m, 3H, ArH), 7.19 (t, 1H, *J* = 8.5 Hz, ArH), 7.15–7.11 (m, 2H, ArH), 6.38 (d, 1H, *J* = 5.1 Hz, ArH), 4.06 (s, 3H, OCH_3_), 4.04 (s, 3H, OCH_3_), 3.71–3.62 (m, 2H, NCH_2_), 2.83–2.76 (m, 1H, CHH), 2.07–1.97 (m, 2H, CH_2_), 1.87–1.81 (m, 1H, CHH), 1.71 (s, 3H, CH_3_); ^13^C-NMR (150 MHz, CDCl_3_) δ 173.7, 170.1, 162.4, 160.8, 160.2, 153.7, 153.0, 149.7, 148.9, 146.9, 138.7, 137.1, 128.2, 123.8, 116.6, 116.5, 116.0, 116.0, 115.6, 109.2, 107.9, 102.3, 99.5, 56.3, 56.2, 53.1, 50.5, 30.9, 27.7, 20.7, 14.3; MS (ESI pos ion) *m/z*: calcd for C_30_H_27_F_2_N_3_O_5_ 547.2, found 548.2 [M + H]^+^; HR-MS (ESI) Calcd for C_30_H_28_F_2_N_3_O_5_ 548.1997 [M + H]^+^, found 548.2012.

*N-(4-((6,7-dimethoxyquinolin-4-yl)oxy)-3-fluorophenyl)-3-ethyl-1-(4-fluorophenyl)-2-oxopiperidine-3-carboxamide* (**20d**, from **14** and **6b**): 58% yield; ^1^H-NMR (600 MHz, CDCl_3_) δ 9.99 (s, 1H, NH), 8.47 (d, 1H, *J* = 5.4 Hz, ArH), 7.82 (dd, 1H, *J* = 12.1, 2.4 Hz, ArH), 7.58 (s, 1H, ArH), 7.44 (s, 1H, ArH), 7.24–7.16 (m, 4H, ArH), 7.15–7.11 (m, 2H, ArH), 6.39 (d, 1H, *J* = 5.2 Hz, ArH), 4.06 (s, 3H, OCH_3_), 4.05 (s, 3H, OCH_3_), 3.71–3.59 (m, 2H, NCH_2_), 2.81 (1H, ddd, *J* = 13.9, 6.4, 2.7 Hz, CHH), 2.22–2.14 (m, 1H, CHH), 2.12–1.93 (m, 4H, CH_2_CH_3_), 1.82–1.76 (m, 1H, CHH), 1.02 (t, 3H, *J* = 7.4 Hz, CH_2_CH_3_); ^13^C-NMR(150 MHz, CDCl_3_) δ 173.3, 169.0, 165.8, 162.5, 160.4, 155.3, 153.7, 153.1, 149.7, 148.7, 146.8, 138.8, 137.1, 137.1, 137.0, 136.9, 128.3, 115.7, 107.8, 102.3, 99.6, 56.3, 55.2, 53.1, 33.8, 27.0, 20.8; MS (ESI pos ion) *m/z*: calcd for C_31_H_29_F_2_N_3_O_5_ 561.2, found 562.2 [M + H]^+^; HR-MS (ESI) Calcd for C_31_H_30_F_2_N_3_O_5_ 562.2154 [M + H]^+^, found 562.2160.

*N-(4-((6,7-dimethoxyquinolin-4-yl)oxy)-3-fluorophenyl)-3-butyl-1-(4-fluorophenyl)-2-oxopiperidine-3-carboxamide* **(20e**, from **14** and **6c**): 64% yield; ^1^H-NMR (600 MHz, CDCl_3_) δ 9.98 (s, 1H, NH), 8.47 (d, 1H, *J* = 5.4 Hz, ArH), 7.82 (dd, 1H, *J* = 12.5, 2.6 Hz, ArH), 7.58 (s, 1H, ArH), 7.42 (s, 1H, ArH), 7.25–7.16 (m, 4H, ArH), 7.16–7.11 (m, 2H, ArH), 6.39 (d, 1H, *J* = 5.1 Hz, ArH), 4.06 (s, 3H, OCH_3_), 4.05 (s, 3H, OCH_3_), 3.71–3.59 (m, 2H, NCH_2_), 2.82 (ddd, 1H, *J* = 14.0, 6.1, 2.7 Hz, CHH), 2.13–1.92 (m, 4H, 2 × CH_2_), 1.84–1.77 (m, 1H, CHH), 1.42–1.28 (m, 4H, 2 × CH_2_), 0.95–0.89 (m, 3H, CH_2_CH_3_); ^13^C-NMR (150 MHz, CDCl_3_) δ 173.3, 169.1, 160.3, 153.0, 149.7, 148.9, 147.0, 138.8, 137.1, 128.3, 128.2, 123.8, 116.7, 116.5, 116.0, 115.7, 109.4, 108.0, 102.3, 99.6, 56.3, 56.3, 54.9, 53.1, 40.5, 27.5, 27.0, 23.0, 20.9, 14.1; MS (ESI pos ion) *m/z*: calcd for C_33_H_33_F_2_N_3_O_5_ 589.2, found 590.2 [M + H]^+^; HR-MS (ESI) Calcd for C_33_H_34_F_2_N_3_O_5_ 590.2467 [M + H]^+^, found 590.2478.

#### 3.2.4. Preparation of **16a** and **16b**

To amide **15a** or **15b** (0.2 mmol) in ethyl acetate (2 mL), acetonitrile (2 mL), and water (1 mL) at 0 °C was added iodobenzene diacetate (82 mg, 0.26 mmol). After stirring at room temperature for 2 h, saturated NaHCO_3_ (3 mL) was added, followed by 30 mL of ethyl acetate. The mixture was filtered, and the filtrate was washed with brine (3 × 5 mL), dried over Na_2_SO_4_ and concentrated *in vacuo*. The residue was purified by flash chromatography on silica gel to give compounds **16a**–**b**.

*N-(4-((2-amino-3-chloropyridin-4-yl)oxy)-3-fluorophenyl)-1-(4-fluorophenyl)-2-oxopiperidine-3-carboxamide* (**16a**). white solid; yield 72%; ^1^H-NMR (600 MHz, CDCl_3_) δ 10.00 (s, 1H, NH), 7.71 (dd, 1H, *J* = 12.6, 2.5 Hz, ArH), 7.24–7.18 (m, 3H, ArH), 7.17–7.10 (m, 3H, ArH), 7.02 (dt, 1H, *J* = 9.0, 1.8 Hz, ArH), 6.62 (t, 1H, *J* = 8.9 Hz, ArH), 5.01 (s, 2H, NH_2_), 3.65 (dq, 2H, *J* = 7.2, 4.3, 3.4 Hz, NCH_2_), 3.54 (t, *J* = 6.3 Hz, 1H, CH), 2.59–2.49 (m, 1H, CHH), 2.21–2.15 (m, 1H, CHH), 2.10–2.05 (m, 1H, CHH), 2.04–1.98 (m, 1H, CHH);^ 13^C-NMR (150 MHz, CDCl_3_) 169.4, 165.7, 162.5, 160.9, 155.6, 154.7, 151.1, 146.5, 139.9, 138.5, 134.5, 134.4, 128.2, 128.2, 123.3, 116.9, 116.8, 116.7, 116.6, 116.6, 115.3, 115.2, 109.4, 109.2, 108.9, 52.8, 47.6, 47.5, 29.8, 22.9, 21.8; MS (ESI pos ion) *m/z*: calcd for C_23_H_19_ClF_2_N_4_O_3_ 472.1, found 473.1 [M + H]^+^; HR-MS (ESI) Calcd for C_23_H_20_ClF_2_N_4_O_3_ 473.1192 [M + H]^+^, found 473.1214.

*N-(4-((2-amino-3-chloropyridin-4-yl)oxy)-3-fluorophenyl)-3-chloro-1-(4-fluorophenyl)-2-oxo-piperidine-3-carboxamide* (**16b**). white solid; yield 76%; ^1^H-NMR (600 MHz, CDCl_3_) δ 10.02 (s, 1H, NH), 7.77 (d, 1H, *J* = 5.8 Hz, ArH), 7.74 (dd, 1H, *J* = 12.0, 2.5 Hz, ArH), 7.26–7.23 (m, 2H, ArH), 7.23–7.20 (m, 1H, ArH), 7.16–7.11 (m, 3H, ArH), 5.99 (dd, 1H, *J* = 5.8, 1.0 Hz, ArH), 5.04 (br s, 2H, NH2), 3.84–3.74 (m, 1H, CHH), 3.74–3.66 (m, 1H, CHH), 2.94–2.82 (m, 1H, CHH), 2.63–2.57 (m, 1H, CHH), 2.43–2.34 (m, 1H, CHH), 2.14–2.09 (m, 1H, CHH); ^13^C-NMR (150 MHz, CDCl_3_) δ 166.9, 164.8, 162.6, 161.0, 160.5, 156.6, 155.0, 153.3, 148.6, 147.8, 146.7, 137.9, 136.2, 127.8, 123.4, 116.7, 116.6, 116.2, 109.6, 109.4, 102.6, 102.1, 91.8, 64.2, 52.7, 33.8, 19.5; MS (ESI pos ion) *m/z*: calcd for C_23_H_18_Cl_2_F_2_N_4_O_3_ 506.1, found 507.1 [M + H]^+^; HR-MS (ESI) Calcd for C_23_H_19_Cl_2_F_2_N_4_O_3_ 507.0802 [M + H]^+^, found 507.0816.

#### 3.2.5. Preparation of **19a** and **19b**

Compound **18a** or **18b** (40 mg, 0.07 mmol) was dissolved in TFA and heated to reflux for 6 h. The solvent was removed and the residue was purified by column chromatography, giving the title compound **19a** or **19b** as a pale yellow solid.

*N-(4-((2-aminopyrimidin-4-yl)oxy)-3-fluorophenyl)-1-(4-fluorophenyl)-2-oxopiperidine-3-carbox-amide* (**19a**). 71% yield; ^1^H-NMR (600 MHz, CDCl_3_) δ 10.20 (s, 1 H, NH), 7.95 (s, 1H, ArH), 7.75 (d, 1H, *J* = 11.8 Hz, ArH), 7.21 (dd, 2H, *J* = 8.7, 4.8 Hz, ArH), 7.18 (d, 1H, *J* = 8.6 Hz, ArH), 7.13 (t, 2H, *J* = 8.2 Hz, ArH), 7.07 (t, 1H, *J* = 8.4 Hz, ArH), 6.49 (s, 1H, ArH), 5.77 (s, 1H, NH), 3.67 (q, 2H, *J* = 6.0 Hz, NCH_2_), 3.57 (s, 1H, CH), 2.57–2.46 (m, 1H, CHH), 2.25–2.16 (m, 1H, CHH), 2.13–1.95 (m, 2H, CH_2_); ^13^C-NMR (150 MHz, CDCl_3_) δ 166.3, 162.5, 160.9, 154.4, 152.8, 138.4, 137.8, 134.1, 128.2, 128.1, 123.1, 116.7, 116.5, 115.6, 108.9, 108.7, 52.7, 47.7, 29.8, 23.0, 21.7; MS (ESI pos ion) *m/z*: calcd for C_22_H_19_F_2_N_5_O_3_ 439.1, found 440.1 [M + H]^+^; HR-MS (ESI) Calcd for C_22_H_20_F_2_N_5_O_3_ 440.1534 [M + H]^+^, found 440.1528.

*N-(4-((2-aminopyrimidin-4-yl)oxy)-3-fluorophenyl)-3-chloro-1-(4-fluorophenyl)-2-oxopiperidine-3-carboxamide* (**19b**). 87% yield; ^1^H-NMR (600 MHz, CDCl_3_) δ 10.04 (s, 1H, NH), 7.96 (d, 1H, *J* = 6.7 Hz, ArH), 7.72 (dd, 1H, *J* = 11.8, 2.5 Hz, ArH), 7.25–7.18 (m, 3H, ArH), 7.16–7.06 (m, 3H, ArH), 6.47 (d, 1H, *J* = 6.7 Hz, ArH), 3.79 (ddd, 1H, *J* = 12.4, 10.1, 4.7 Hz, CHH), 3.69 (dt, 1H, *J* = 11.3, 4.5 Hz, CHH), 2.88 (ddd, 1H, *J* = 14.9, 11.6, 3.1 Hz, CHH), 2.63–2.49 (m, 1H, CHH), 2.44–2.31 (m, 1H, CHH), 2.15–2.05 (m, 1H, CHH); ^13^C-NMR (150 MHz, CDCl_3_) δ 171.2, 166.8, 164.9, 162.6, 161.0, 154.5, 152.8, 137.9, 137.9, 137.2, 137.1, 134.8, 134.7, 127.9, 127.9, 127.8, 123.3, 116.7, 116.6, 116.0, 109.3, 109.2, 109.1, 109.1, 100.0, 99.0, 64.4, 52.7, 33.8, 19.4; MS (ESI pos ion) *m/z*: calcd for C_22_H_18_ClF_2_N_5_O_3_ 473.1, found 474.0 [M + H]^+^; HR-MS (ESI) Calcd for C_22_H_19_ClF_2_N_5_O_3_ 474.1145 [M + H]^+^, found 474.1151.

### 3.3. Biology

#### 3.3.1. c-Met Kinase Assay

The effects of indicated compound on the activities of c-Met kinases were determined using enzyme-linked immunosorbent assays (ELISAs) with purified recombinant proteins. Briefly, 20 μg/mL poly (Glu,Tyr)_4:1_ (Sigma, St. Louis, MO, USA) was pre-coated in 96-well plates as a substrate. A 50-μL aliquot of 10 μmol/L ATP solution diluted in kinase reaction buffer (50 mmol/L HEPES [pH 7.4], 50 mmol/L MgCl_2_, 0.5 mmol/L MnCl_2_, 0.2 mmol/L Na_3_VO_4_, and 1 mmol/L DTT) was added to each well; 1 μL of various concentrations of indicated compound diluted in 1% DMSO (*v/v*) (Sigma) were then added to each reaction well. DMSO (1%, *v/v*) was used as the negative control. The kinase reaction was initiated by the addition of purified c-Met tyrosine kinase proteins diluted in 49 μL of kinase reaction buffer. After incubation for 60 min at 37 °C, the plate was washed three times with phosphate-buffered saline (PBS) containing 0.1% Tween 20 (T-PBS). Anti-phosphotyrosine (PY99) antibody (100 μL; 1:500, diluted in 5 mg/mL BSA T-PBS) was then added. After a 30-min incubation at 37 °C, the plate was washed three times, and 100 μL horseradish peroxidase-conjugated goat anti-mouse IgG (1:2000, diluted in 5 mg/mL BSA T-PBS) was added. The plate was then incubated at 37 °C for 30 min and washed 3 times. A 100-μL aliquot of a solution containing 0.03% H_2_O_2_ and 2 mg/ml *o*-phenylenediamine in 0.1 mol/L citrate buffer (pH 5.5) was added. The reaction was terminated by the addition of 50 μL of 2 mol/L H_2_SO_4_ as the color changed, and the plate was analyzed using a multi-well spectrophotometer (SpectraMAX 190, Molecular Devices, Sunnyvale, CA, USA) at 490 nm. The inhibition rate (%) was calculated using the following equation: [1 − (A490/A490 control)] × 100%. The IC_50_ values were calculated from the inhibition curves in two separate experiments.

#### 3.3.2. Cell Proliferation Assay

Cells were seeded in 96-well tissue culture plates. On the next day, the cells were exposed to various concentrations of compounds and further cultured for 72 h. Cell proliferation was then determined using sulforhodamine B (SRB, Sigma, St. Louis, MO, USA). The IC_50 _values were calculated by concentration-response curve fitting using the four-parameter method.

## 4. Conclusions

In summary, a series of compounds based upon the 3-carboxylpiperidin-2-one scaffold were designed, synthesized and evaluated for their c-Met kinase inhibition and cytotoxicity against MKN45 cancer cell lines. Five compounds (**16b**, **20b**–**e**) exhibited moderate to excellent activity against c-Met kinase, with IC_50_ values ranging from 8.6–81 nM. Moreover, four compounds (**20b**–**e**) showed potent inhibitory activity against MKN45 cell proliferation, with IC_50_s ranging from 0.57–16 μM. Further structure-activity relationship studies are under way in our laboratory and will be reported in due course.
